# Cytotoxicity and Characterization of Zinc Oxide and Silver Nanoparticles Synthesized Using Ocimum tenuiflorum and Ocimum gratissimum Herbal Formulation

**DOI:** 10.7759/cureus.53481

**Published:** 2024-02-03

**Authors:** Remmiya Mary Varghese, Aravind Kumar S, Rajeshkumar Shanmugam

**Affiliations:** 1 Orthodontics and Dentofacial Orthopedics, Saveetha Dental College and Hospitals, Saveetha Institute of Medical and Technical Sciences, Saveetha University, Chennai, IND; 2 Nano-Biomedicine Lab, Center for Global Health Research, Saveetha Medical College and Hospitals, Saveetha Institute of Medical and Technical Sciences, Saveetha University, Chennai, IND; 3 Pharmacology, Saveetha Dental College and Hospitals, Saveetha Institute of Medical and Technical Sciences, Saveetha University, Chennai, IND

**Keywords:** ocimum gratissimum, ocimum tenuiflorum, green synthesis, black tulsi, african basil, znonps, agnps

## Abstract

Background

Toxicological assessments of nanoparticles are becoming more and more necessary due to the current rapid increase in interest in them for biomedical applications. This study aimed to synthesize and characterize zinc oxide nanoparticles (ZnONPs) and silver nanoparticles (AgNPs) using *Ocimum tenuiflorum* (black tulsi) and *Ocimum gratissimum* (African basil) herbal formulation extracts and to evaluate their cytotoxic effects.

Methods

The synthesis of AgNPs and ZnONPs was monitored using UV-visible spectra analysis at different time intervals. The nanoparticles' morphology and elemental composition were examined via scanning electron microscopy (SEM) and energy-dispersive X-ray spectroscopy (EDX). Furthermore, Fourier-transform infrared spectroscopy (FT-IR) spectra analysis was employed to identify the functional groups within the nanoparticles. The cytotoxic effects of the nanoparticles were evaluated using the brine shrimp lethality assay.

Results

The UV-visible spectra analysis revealed the successful synthesis of AgNPs and ZnONPs, with maximum absorption peaks observed at 430 nm and 380 nm, respectively. SEM images showed that AgNPs were spherical in shape and tended to agglomerate, while ZnONPs displayed a unique rod-like to short prism shape, and EDX analysis confirmed the presence of both silver and zinc in these nanoparticles, alongside other elements from the herbal extracts. FT-IR analysis indicated the existence of diverse functional groups on the nanoparticles' surfaces. The brine shrimp lethality assay results demonstrated a concentration-dependent cytotoxic effect of the nanoparticles.

Conclusion

The study successfully synthesized and characterized AgNPs and ZnONPs using *Ocimum tenuiflorum* and *Ocimum gratissimum* herbal formulation extracts. The nanoparticles exhibited significant cytotoxic effects, suggesting their potential applications in various fields. Our results highlight the need for a more discrete use of nanoparticles for biomedical applications. Further studies are needed to explore their potential uses and ensure their safe and effective application.

## Introduction

Nanotechnology, which involves the manipulation of matter at the atomic and molecular scale, has emerged as a significant field of research with potential applications across various sectors, including medicine, electronics, biomaterials, and energy production [1,2]. Silver nanoparticles (AgNPs) and zinc oxide nanoparticles (ZnONPs) have attracted significant attention among the various types of nanoparticles due to their distinct physical, chemical, and biological properties [3].

AgNPs are well-known for their exceptional antibacterial, antifungal, antiviral, anti-inflammatory, and anticancer properties [3,4]. They have found applications in various fields, including wound dressings, coatings for medical devices, water treatment, and biosensing [4]. Conversely, ZnONPs are renowned for their UV-blocking capabilities, antimicrobial activity, and potential utility in cancer therapy [5]. They have been widely employed in products such as sunscreens, cosmetics, paints, and coatings [6].

Traditionally, nanoparticles have been synthesized through physical and chemical methods, which often involve high energy consumption, the use of toxic chemicals, and the generation of hazardous by-products [7]. As a result, there is a rising interest in the advancement of eco-friendly and sustainable green synthesis techniques. These methods harness biological entities such as plants, bacteria, fungi, and algae for nanoparticle synthesis [8]. This method is advantageous due to its cost-effectiveness, eco-friendliness, and the fact that it does not necessitate high pressure, energy, temperature, or toxic chemicals [9].

In this context, the utilization of medicinal plants for nanoparticle synthesis has gained substantial attention. Medicinal plants are rich sources of bioactive compounds that can serve as both reducing and capping agents in the nanoparticle synthesis process [10]. African basil (*Ocimum gratissimum*) [11] and black tulsi (*Ocimum tenuiflorum*) [12], also known as holy basil, are well-known for their therapeutic properties, which encompass antimicrobial, anticancer, anti-inflammatory, and antioxidant activities. These properties can be attributed to the presence of various bioactive compounds, such as phenolics, flavonoids, terpenoids, and alkaloids, which can facilitate nanoparticle synthesis [13].

The primary objective of this study is to synthesize and characterize AgNPs and ZnONPs using herbal formulation extracts derived from *Ocimum gratissimum* and *Ocimum tenuiflorum*. The synthesized nanoparticles will be characterized using multiple analytical techniques: UV-visible spectra analysis, energy-dispersive X-ray spectroscopy (EDX), scanning electron microscopy (SEM), and Fourier-transform infrared spectroscopy (FTIR). Additionally, the cytotoxic effects of these nanoparticles will be assessed through the brine shrimp lethality assay. This research offers valuable insights into the potential of medicinal plants for environmentally friendly nanoparticle synthesis and their diverse applications across various fields.

## Materials and methods

Preparation of herbal formulation

About 1 g of *Ocimum tenuiflorum* and 1 g of *Ocimum gratissimum* were accurately added to 100 mL of distilled water. The mixture was then subjected to heating using a heating mantle at a temperature of 60 degrees Celsius for a duration of 15-20 minutes. Subsequently, the boiled mixture underwent a gradual filtration process utilizing filter paper. The resulting filtrate, containing the extract, was then stored for subsequent nanoparticle synthesis.

Green synthesis of ZnONPs and AgNPs

The synthesis of ZnONPs using a green approach involving *Ocimum tenuiflorum* and *Ocimum gratissimum* extracts in the presence of zinc nitrate solution (30 mm in 50 ml distilled water) was undertaken in this study. This environmentally friendly method harnesses the bioactive compounds present in these herbal extracts for the reduction and stabilization of ZnONPs. The procedure began with the preparation of a zinc nitrate solution, providing a controlled source of zinc ions. Subsequently, 50 ml of *Ocimum tenuiflorum* and *Ocimum gratissimum* extracts, known for their rich phytochemical content, were combined with the zinc nitrate solution. Similarly, for the green synthesis of AgNPs, a 1 mm silver nitrate solution was prepared by dissolving silver nitrate in 80 mL of distilled water. To this solution, 20 mL of a filtered herbal formulation extract was added. The resulting mixtures were subjected to a centrifugation process at 8000 rpm for 10 minutes.

The centrifugation step played a pivotal role in both the ZnONP and AgNP synthesis processes. It facilitated the separation of synthesized nanoparticles from any unreacted precursors or extract residues. The pellet collected after centrifugation represents the desired ZnONPs and AgNPs, which were then subjected to further characterization and evaluation.

Characterization and cytotoxic effect

The ZnONPs and AgNPs synthesized through the green method were characterized using UV-visible spectra analysis, FT-IR, SEM, and EDX. The brine shrimp lethality assay was conducted using green-synthesized ZnONPs and AgNPs. Brine shrimp eggs were hatched, and the nauplii were collected. Stock solutions of nanoparticles were prepared and diluted to concentrations of 5, 10, 20, 40, and 80 μg/mL. In 6-well ELISA plates, 10 nauplii were exposed to each nanoparticle concentration for 24 and 48 hours. Live nauplii were counted, and the percentage of survival was determined. The LC50, the concentration at which 50% of nauplii were killed, was calculated. This assay provides an efficient method to assess the cytotoxicity of green-synthesized nanoparticles, offering insights into their potential applications and safety.

## Results

UV vis spectroscopy

**Figure 1:**
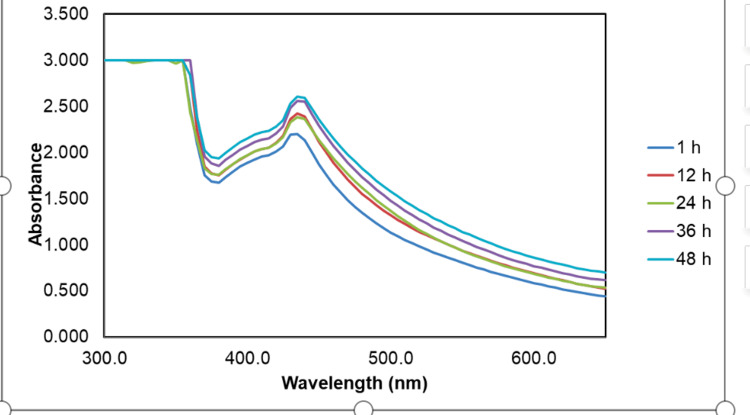
UV-visible spectra of green synthesized AgNPs recorded at different time intervals (one hour, 12 hours, 24 hours, 36 hours, and 48 hours) to investigate the time-dependent changes The UV-visible spectra analysis revealed the successful synthesis of AgNPs with maximum absorption peaks observed at 430 nm. AgNPs: silver nanoparticles

In Figure 1, the UV-visible spectra analysis of the herbal formulation consisting of *Ocimum tenuiflorum *and *Ocimum gratissimum-*mediated AgNPs was observed to have a maximum absorption peak of 430 nm. Spectra were recorded at different time intervals (one hour, 12 hours, 24 hours, 36 hours, and 48 hours) to investigate the time-dependent changes. At one hour, a subtle absorption peak at 430 nm indicated the early formation of AgNPs, intensifying significantly at 12 hours and reaching a stable state by 24 hours, suggesting the completion of AgNP synthesis. Further measurements at 36 and 48 hours showed no substantial changes in the absorption peak, suggesting a state of equilibrium. Notably, there were no discernible differences observed in the spectra over time, highlighting the consistency of the AgNP synthesis process using the *Ocimum tenuiflorum* and *Ocimum gratissimum* herbal formulation extracts.

**Figure 2:**
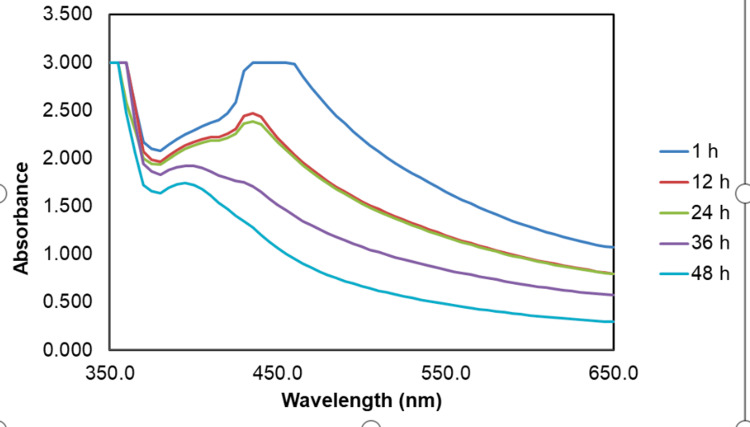
UV-visible spectra of Ocimum tenuiflorum and Ocimum gratissimum herbal formulation-mediated ZnONPs recorded at different time intervals (1 hour, 12 hours, 24 hours, 36 hours, and 48 hours) to investigate the time-dependent changes The UV-visible spectra analysis revealed the successful synthesis of ZnONPs, with maximum absorption peaks observed at 380 nm. ZnONPs: zinc oxide nanoparticles

Figure 2 displays the UV-visible spectra of ZnONPs synthesized using the *Ocimum tenuiflorum* and *Ocimum gratissimum* herbal formulation extracts, with a maximum absorption peak observed at approximately 380 nm, indicating the presence of ZnONPs. Time-dependent analysis at one hour showed an absorption peak at 450 nm, while at 12 and 24 hours, the peak shifted to 440 nm, suggesting ongoing ZnONP formation. Interestingly, at 36 and 48 hours, the absorption peak stabilized at 380 nm, indicating the maturation and stability of ZnONPs. This shift in peak over time suggests dynamic changes in the optical properties, potentially related to nanoparticle size or aggregation during the synthesis process.

SEM analysis of AgNPs and ZnONPs

**Figure 3:**
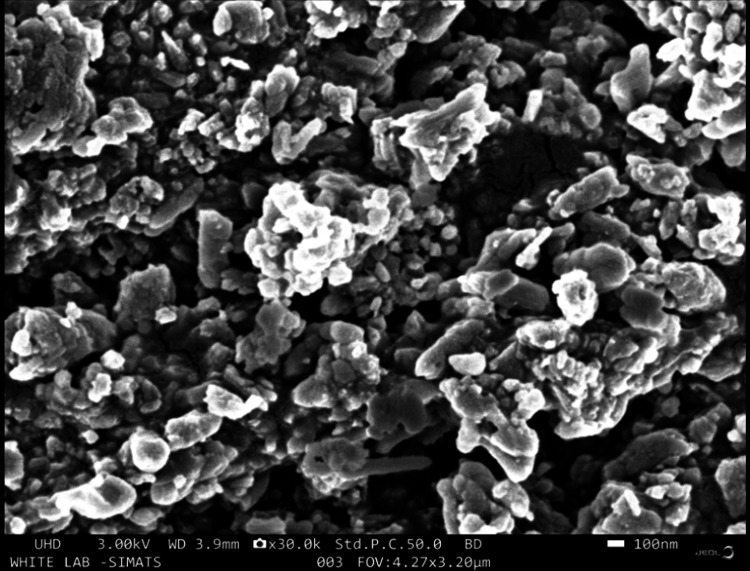
SEM image of green-synthesized AgNPs AgNP morphology was examined via SEM. SEM images showed that the AgNPs were spherical in shape and tended to agglomerate. AgNPs: silver nanoparticles, SEM: scanning electron microscopy

Figure 3 presents SEM images of AgNPs synthesized using a green synthesis approach. The images reveal the morphological characteristics of the AgNPs. The AgNPs appear to be spherical in shape, and they exhibit a tendency to agglomerate. These results indicate that the green synthesis method used in this study resulted in the creation of spherical AgNPs, likely due to the presence of reducing and stabilizing agents in the synthesis process. The observed agglomeration of nanoparticles could be a result of their natural tendency to cluster due to interparticle forces or surface properties. Overall, the SEM images provide valuable insights into the physical attributes of the synthesized AgNPs, indicating their suitability for various applications.

**Figure 4:**
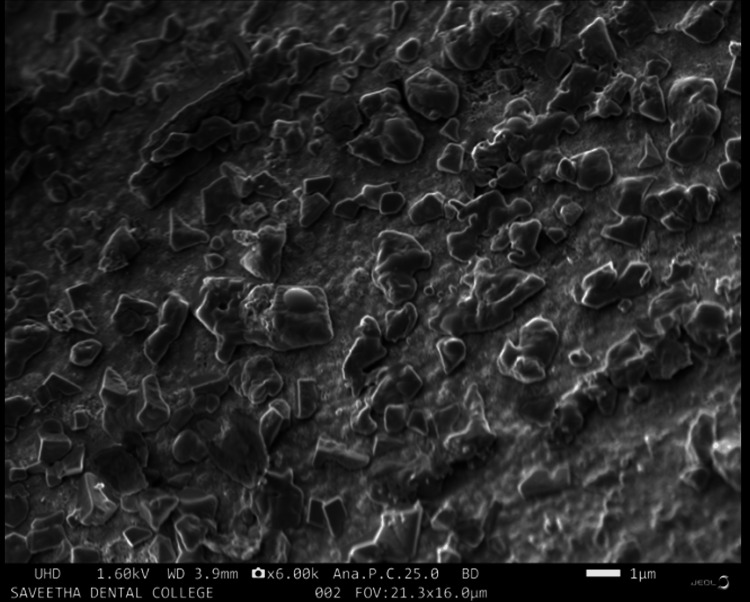
SEM image of green-synthesized ZnONPs ZnONP morphology was examined via SEM. SEM images showed that the ZnONPs displayed a unique rod-like to short prism shape. ZnONPs: zinc oxide nanoparticles, SEM: scanning electron microscopy

In Figure 4, SEM imaging of the ZnONPs provides a comprehensive view of their morphology. The ZnONPs are observed to exhibit a distinctive morphological structure, appearing as rod-like to short prism shapes. This morphological variation suggests the presence of different crystalline facets or growth patterns during the synthesis process. The rod-like and prism-like structures are indicative of the versatile morphological features that can be achieved through green synthesis methods. Such morphological diversity is of significant interest in various applications, as it can influence the physical and chemical properties of ZnONPs, making them suitable for a wide range of technological and biomedical applications. The SEM image underscores the successful green synthesis of ZnONPs with the desired morphology, presenting a promising avenue for further exploration and utilization in various fields.

EDX analysis of AgNPs

**Figure 5:**
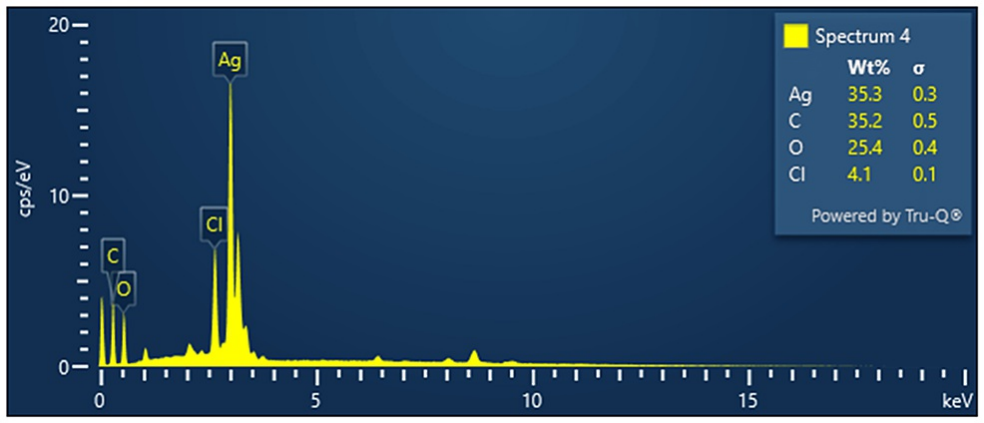
EDX spectra of Ocimum tenuiflorum and Ocimum gratissimum herbal formulation-based AgNPs AgNP elemental composition was examined via EDX. The predominant element detected is silver (Ag), constituting approximately 35.3% of the elemental composition. AgNPs: silver nanoparticles, EDX: energy-dispersive X-ray spectroscopy

The EDX spectra obtained from the analysis of *Ocimum tenuiflorum *and *Ocimum gratissimum*-based AgNPs depicted in Figure 5 reveal the elemental composition of the synthesized nanoparticles. In both samples, the predominant element detected is silver, constituting approximately 35.3% of the elemental composition. Carbon is also present prominently, accounting for approximately 35.2% of the composition. Oxygen is detected at a concentration of 25.4%, while chlorine is observed at 4.1%. These findings indicate a substantial presence of silver and carbon, which may be attributed to the organic compounds derived from the *Ocimum tenuiflorum *and *Ocimum gratissimum* herbal extracts used in the synthesis process. The presence of oxygen suggests the presence of oxides or surface modifications on the AgNPs, and the detection of chlorine may be linked to the synthesis process.

**Figure 6:**
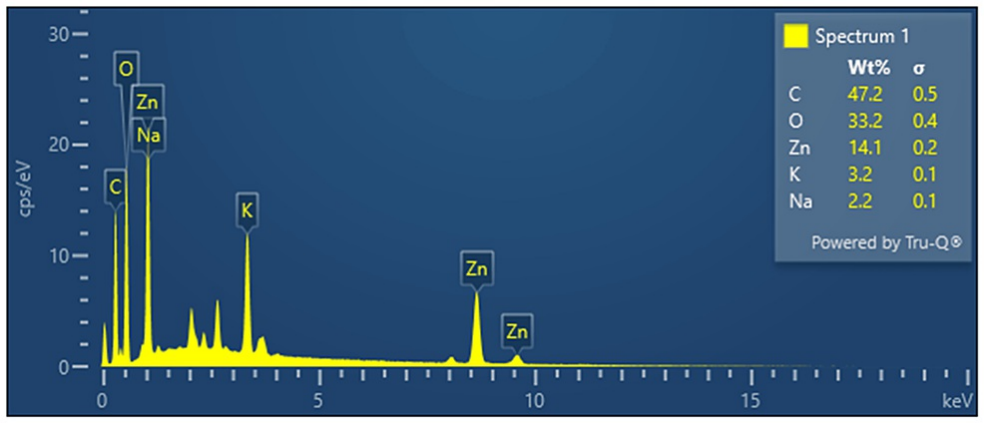
EDX spectra of Ocimum tenuiflorum and Ocimum gratissimum herbal formulation-mediated ZnONPs ZnONP elemental composition was examined via EDX. It confirmed the presence of zinc in these nanoparticles, alongside other elements from the herbal extracts. ZnONPs: zinc oxide nanoparticles, EDX: energy-dispersive X-ray spectroscopy

In Figure 6, the EDX spectra obtained from the analysis of ZnONPs synthesized using *Ocimum tenuiflorum* and *Ocimum gratissimum* reveal the elemental composition of the synthesized nanoparticles. Carbon is detected as the most abundant element, comprising approximately 47.2% of the composition, likely originating from organic compounds within the herbal extracts. Oxygen constitutes 33.2% of the composition, indicating the presence of oxides or surface modifications on the ZnONPs. Zinc, the intended element for synthesis, is observed at a concentration of 14.1%, confirming the successful formation of ZnONPs. Additionally, smaller percentages of potassium at 3.2% and sodium at 2.2% are detected, possibly stemming from herbal extracts or other sources. These findings provide insights into the elemental composition of the ZnONPs synthesized through the *Ocimum tenuiflorum *and *Ocimum gratissimum*-mediated green synthesis processes, highlighting the influence of the herbal extracts on their composition.

FT-IR spectroscopy of AgNPs and ZnONPs

**Figure 7:**
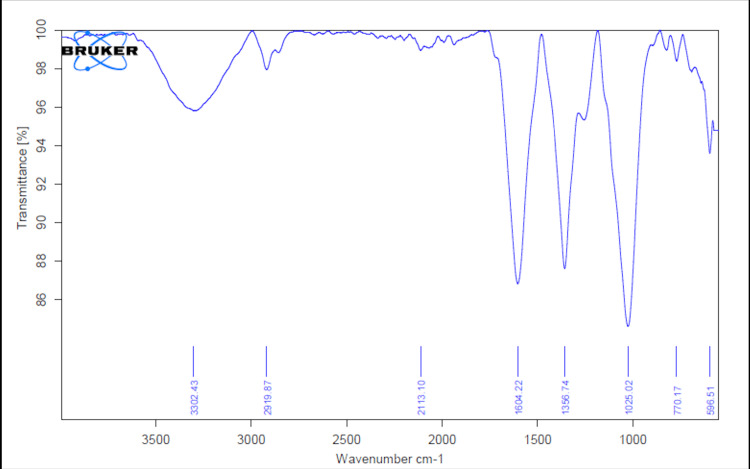
FT-IR spectra of green-synthesized AgNPs FT-IR spectra analysis was employed to identify the functional groups within the nanoparticles. AgNPs: silver nanoparticles, FT-IR: Fourier-transform infrared spectroscopy

In Figure 7, the FT-IR spectra analysis of green-synthesized AgNPs revealed several distinctive peaks at specific wavenumbers: 3302.43 cm-1, 2919.87 cm-1, 2113.10 cm-1, 1604.22 cm-1, 1356.74 cm-1, 1025.02 cm-1, 770.17 cm-1, and 596.51 cm-1. The broad absorption band at 3302.43 cm-1 suggests the presence of hydroxyl groups on the AgNP surface, likely stemming from the bio-reduction process involved in green synthesis. The peak at 2919.87 cm-1 corresponds to C-H stretching vibrations, indicating the involvement of organic molecules in the reduction and stabilization of AgNPs. The 2113.10 cm-1 peak, though unusual, warrants further investigation for its potential chemical significance. The presence of C=O stretching vibrations at 1604.22 cm-1 suggests the existence of carbonyl groups, possibly originating from molecules involved in capping or stabilizing the AgNPs. Moreover, the absorption band at 1356.74 cm-1 indicates C-N stretching vibrations, possibly related to amino groups or other nitrogen-containing compounds. Peaks at 1025.02 cm-1, 770.17 cm-1, and 596.51 cm-1 may represent additional functional groups or vibrational modes and require further scrutiny to determine their specific chemical associations.

**Figure 8:**
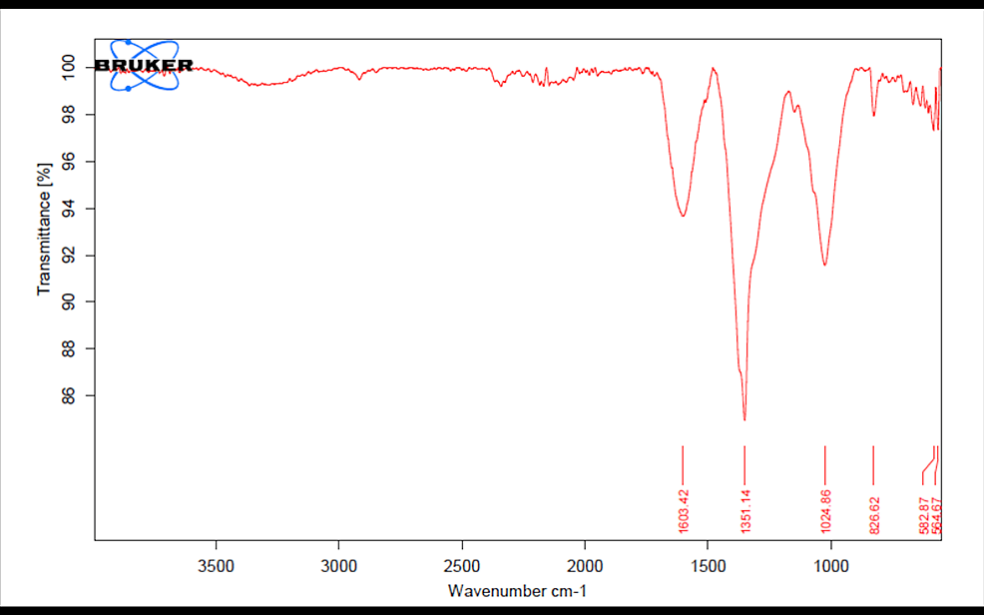
FT-IR spectra of green-synthesized ZnONPs FT-IR spectra analysis was employed to identify the functional groups within the nanoparticles. ZnONPs: zinc oxide nanoparticles, FT-IR: Fourier-transform infrared spectroscopy

In Figure 8, the FT-IR spectra analysis of ZnONPs revealed distinct peaks at specific wavenumbers: 1603.42 cm-1, 1351.14 cm-1, 1024.86 cm-1, 826.62 cm-1, 582.87 cm-1, and 564.67 cm-1. The dominant peak observed at 1603.42 cm-1 signifies an absorption band typically linked to the stretching vibrations of C=O bonds, implying the presence of carbonyl groups on the surface of ZnONPs. These carbonyl groups are likely associated with organic molecules engaged in the green synthesis procedure, potentially contributing to the nanoparticles' stabilization. Additionally, the peak at 1351.14 cm-1 suggests the presence of C-N stretching vibrations, which may indicate the existence of amino groups or other nitrogen-containing compounds on the surface of the ZnONPs. The absorption band observed at 1024.86 cm-1 is likely associated with the stretching vibrations of Zn-O bonds, which are characteristic of zinc oxide compounds. This peak provides strong evidence of the formation of ZnONPs during the green synthesis process. The peaks at 826.62 cm-1, 582.87 cm-1, and 564.67 cm-1 may represent additional functional groups or vibrational modes on the ZnONP surface. Further investigation is needed to elucidate the specific chemical nature and significance of these peaks. Overall, the FT-IR spectra of green-synthesized ZnONPs indicate the presence of various functional groups, including carbonyl and amino groups, as well as characteristic Zn-O bonds. These functional groups are likely derived from the organic precursors used in the synthesis and play a crucial role in stabilizing the ZnONPs.

Cytotoxic effect

**Figure 9:**
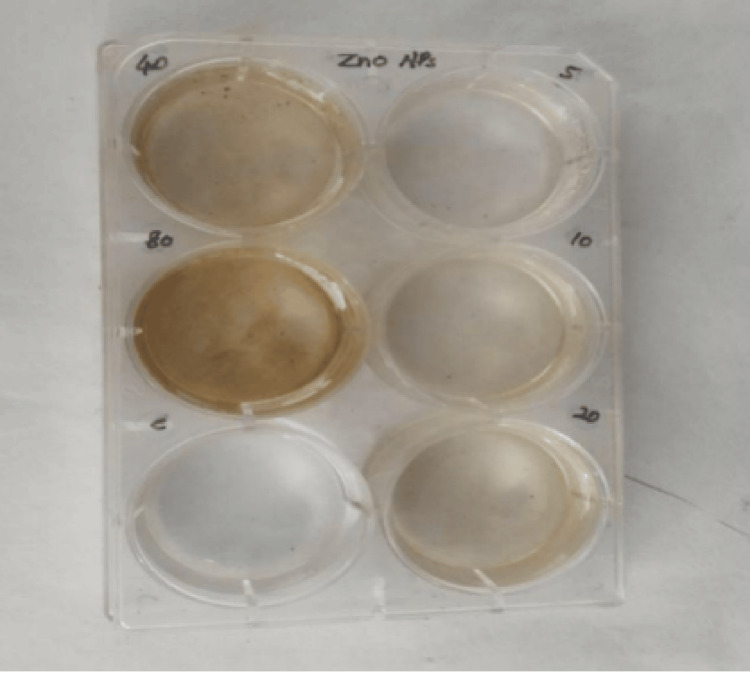
Cytotoxic effect of ZnONPs was performed by brine shrimp lethality assay indicating a concentration-dependent cytotoxic effect of ZnONPs The cytotoxic effect of ZnONPs was assessed using the brine shrimp lethality assay, with the percentage of live nauplii calculated at 20 μg, 40 μg, and 80 μg concentrations of silver nanoparticles. ZnONPs: zinc oxide nanoparticles

**Figure 10:**
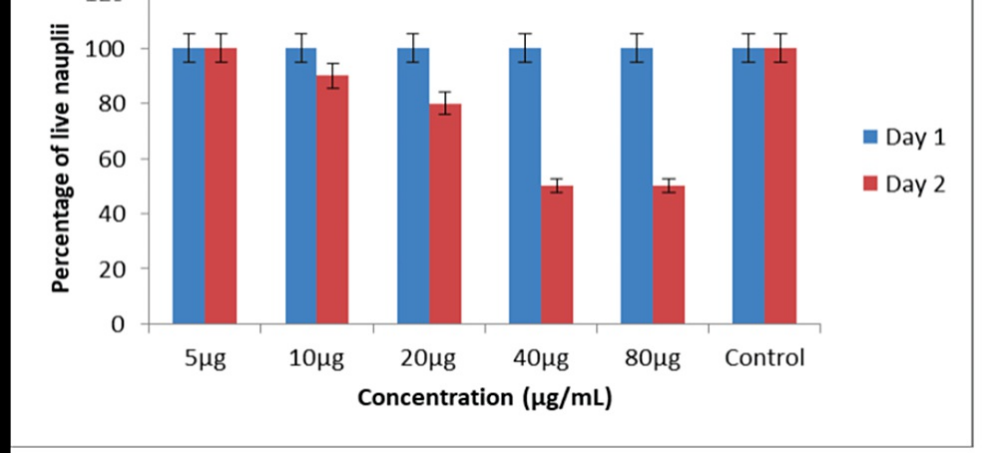
Cytotoxic effect of Ocimum tenuiflorum and Ocimum gratissimum herbal formulation-mediated ZnONPs with the percentage of live nauplii calculated at different ZnONP concentrations on Day 1 and Day 2 which were not statistically significant. The X-axis represents the different ZnONP concentrations on Day 1 and Day 2, whereas the Y-axis represents the percentage of live nauplii at different ZnONP concentrations The cytotoxic effect of ZnONPs was assessed using the brine shrimp lethality assay, with the percentage of live nauplii calculated at 20 μg, 40 μg, and 80 μg concentrations of AgNPs on Day 1 and Day 2. ZnONPs: zinc oxide nanoparticles, AgNPs: silver nanoparticles

The cytotoxic effect of ZnONPs was evaluated using the brine shrimp lethality assay, as depicted in the provided Figures 9-10. The assay results were presented in terms of the percentage of live nauplii at different ZnONP concentrations on Day 1 and Day 2. The brine shrimp lethality assay results indicate a concentration-dependent cytotoxic effect of ZnONPs. On Day 1, at the lowest tested concentration of 5 μg, there were 100% live nauplii, which remained consistent with the control group. On Day 2, the cytotoxic effect of ZnONPs was sustained, with a reduction in survival rates compared to the control group. Notably, at higher concentrations of 40 μg and 80 μg, the percent of live nauplii rate remained at 50%, indicating a potential plateau effect where further increases in concentration did not result in a proportional increase in cytotoxicity.

**Figure 11:**
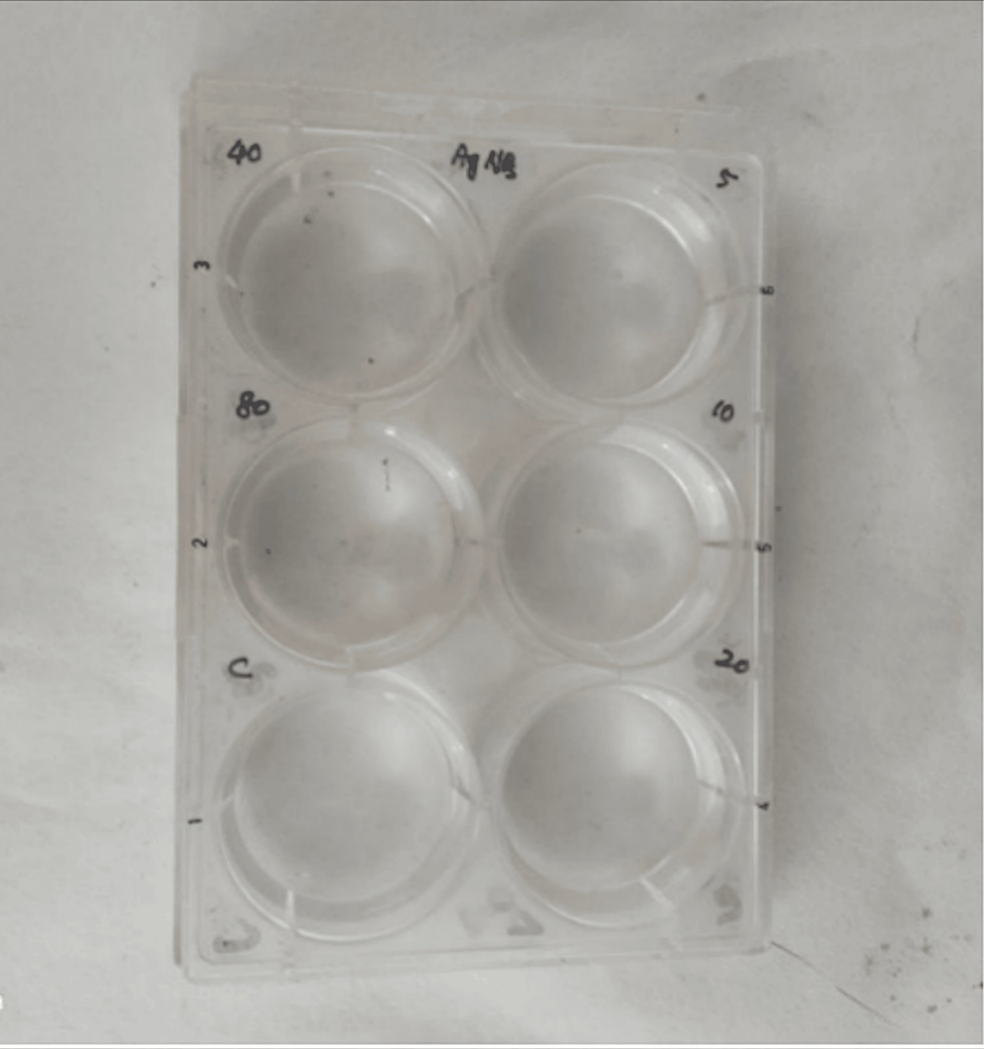
Cytotoxic effect of AgNPs was performed by brine shrimp lethality assay with the percentage of live nauplii calculated at different AgNP concentrations The cytotoxic effect of AgNPs was assessed using the brine shrimp lethality assay, with the percentage of live nauplii calculated at 20 μg, 40 μg, and 80 μg concentrations of AgNPs on Day 1. AgNPs: silver nanoparticles

**Figure 12:**
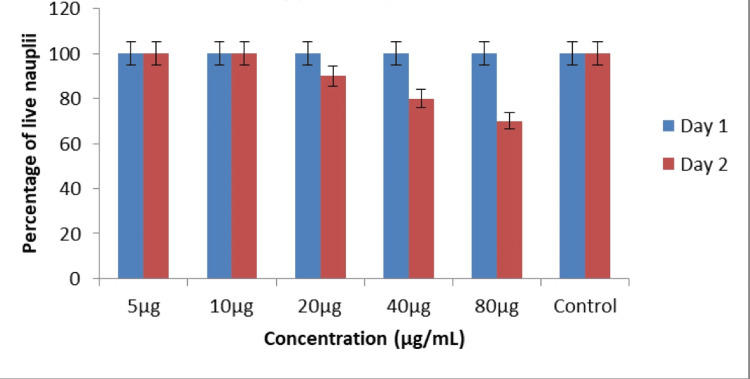
Cytotoxic effect of Ocimum tenuiflorum and Ocimum gratissimum herbal formulation-mediated AgNPs with the percentage of live nauplii calculated at different AgNP concentrations on Day 1 and Day 2 which were not statistically significant. The X-axis represents the different AgNP concentrations on Day 1 and Day 2, whereas the Y-axis represents the percentage of live nauplii at different AgNP concentrations The cytotoxic effect of AgNPs was assessed using the brine shrimp lethality assay, with the percentage of live nauplii calculated at 20 μg, 40 μg, and 80 μg concentrations of AgNPs on Day 1 and Day 2. AgNPs: silver nanoparticles

In Figures 11-12, the cytotoxic effect of AgNPs was assessed using the brine shrimp lethality assay, with the percentage of live nauplii calculated at different AgNP concentrations on both Day 1 and Day 2. The brine shrimp lethality assay results for AgNPs indicate that, at the concentrations tested, there is no significant cytotoxic effect observed on Day 1. The percentage of live nauplii in all AgNP treatment groups and the control group was 100%, suggesting that, at these concentrations, AgNPs did not induce acute lethality within the first day of exposure.

However, on Day 2, a concentration-dependent cytotoxic effect becomes evident. At 20 μg, 40 μg, and 80 μg concentrations of AgNPs, there is a reduction in the percentage of live nauplii compared to the control group, indicating increasing cytotoxicity. This effect becomes more pronounced as the concentration of AgNP increases, with the highest concentration of 80 μg resulting in a noticeable decrease in survival, as reflected by a reduction to 70% live nauplii on Day 2.

## Discussion

The study investigated the cytotoxic effects and characterized the AgNPs and ZnONPs synthesized using a green approach involving *Ocimum tenuiflorum* and *Ocimum gratissimum*. The results encompass a comprehensive analysis of nanoparticle formation, their morphological characteristics, and their potential cytotoxicity. In the UV-visible spectra analysis of the AgNPs synthesized with the herbal formulation, a prominent absorption peak at 430 nm indicated the presence of AgNPs. Time-dependent measurements demonstrated the gradual formation of AgNPs, reaching stability at 24 hours. The consistency of the spectra over time underscored the reliability of the synthesis method [14]. For ZnONPs, a maximum absorption peak at approximately 380 nm confirmed their presence, with a shift in peak observed during synthesis, suggesting dynamic changes in optical properties during formation [15].

SEM images of AgNPs revealed a spherical morphology with a tendency to agglomerate. This morphology is indicative of the green synthesis method and the presence of reducing and stabilizing agents in the process. The EDX spectra displayed the elemental composition, with silver and carbon as predominant elements, suggesting the influence of herbal extracts. In the case of ZnONPs, SEM images revealed distinctive rod-like to short prism shapes, highlighting the versatility of morphology achievable through green synthesis [16]. The EDX spectra validated the existence of zinc, carbon, and oxygen, in alignment with the synthesis process. The FT-IR spectra of AgNPs indicated the presence of various functional groups, such as hydroxyl, carbonyl, and amino groups, likely originating from the herbal extracts. Similarly, the FT-IR spectra of ZnONPs showed the presence of carbonyl and amino groups, as well as characteristic Zn-O bonds.

The cytotoxicity of AgNPs and ZnONPs was evaluated using the brine shrimp lethality assay, which is a valuable preliminary screening tool for toxicity assessment. In cytotoxicity assessments using the brine shrimp lethality assay, AgNPs displayed a concentration-dependent cytotoxic effect on Day 2, with higher concentrations leading to increased mortality. In contrast, ZnONPs exhibited cytotoxicity from Day 2 onwards, with the highest concentration inducing a noticeable decrease in survival. These results emphasize the potential cytotoxicity of both nanoparticles and the importance of concentration and exposure duration in assessing their biological effects. In previous research work, AgNPs synthesized from *Cassia oleoresin* exhibited notably low cytotoxicity at lower concentrations, suggesting their potential for biomedical applications [17]. Furthermore, the versatility of the brine shrimp lethality assay extends beyond nanoparticle assessment. Researchers have leveraged this cost-effective and efficient method to evaluate the in vivo toxicity of novel metallodrugs, including Schiff base metal complexes and cobalt metal complexes, as well as their hydrogel formulations [18]. This demonstrates the assay's adaptability in assessing a wide range of potential toxicants. In addition to nanoparticle studies and metallodrugs, the brine shrimp assay has been employed to explore the cytotoxic properties of natural compounds derived from plant extracts. For instance, the combination of aqueous and ethanolic extracts from *Annona reticulata*, along with *Allium fistolisum *and *Brassica oleracea*, was assessed using this assay, demonstrating strong cytotoxicity against brine shrimp larvae [19,20]. This application underscores the versatility of the brine shrimp lethality assay for assessing the cytotoxic effects of various natural products.

In summary, the study successfully synthesized AgNPs and ZnONPs using *Ocimum tenuiflorum* and *Ocimum gratissimum* and characterized their physicochemical properties. Additionally, it revealed the concentration-dependent cytotoxic effects of both nanoparticles, highlighting the need for further investigations into their potential applications and safety profiles. The combination of green synthesis and cytotoxicity assessments provides valuable insights into the potential use of these nanoparticles in various fields, including medicine, nanotechnology, and environmental science.

Limitations

While this study contributes valuable insights into the synthesis, characterization, and cytotoxic effects of ZnONPs and AgNPs using *Ocimum tenuiflorum* and *Ocimum gratissimum* herbal extracts, several limitations should be considered. Firstly, the study primarily relies on in vitro cytotoxicity assessment using the brine shrimp lethality assay, which, while indicative, may not fully capture the complexities of biological responses. Moreover, the investigation focuses on the cytotoxic effects, overlooking potential long-term impacts, genotoxicity, and immunotoxicity. The study's scope is further constrained by the exclusive use of *Ocimum tenuiflorum* and *Ocimum gratissimum*, limiting the generalizability of the findings to other botanical sources. Additionally, the absence of in vivo studies and environmental impact assessments raises questions about the nanoparticles' safety in living organisms and ecosystems. Furthermore, the suggested applications lack specificity, necessitating more targeted investigations into the nanoparticles' efficacy and safety in specific medical and environmental contexts. Addressing these limitations in future research would enhance the comprehensive understanding of the synthesized nanoparticles and their potential applications.

## Conclusions

This study effectively synthesized and comprehensively characterized AgNPs and ZnONPs using an herbal formulation extract derived from *Ocimum tenuiflorum* and *Ocimum gratissimum*. The UV-visible spectra analysis confirmed the successful synthesis of these nanoparticles, with distinct absorption peaks observed at 430 nm and 380 nm for AgNPs and ZnONPs, respectively. Morphological analysis via SEM revealed spherical AgNPs that tended to agglomerate and distinctive rod-like to short prism-shaped ZnONPs. EDX spectra analysis confirmed the presence of silver and zinc, along with other elements from the herbal extracts. Additionally, FT-IR spectra analysis indicated the presence of various functional groups on the nanoparticle surfaces. Most importantly, the brine shrimp lethality assay demonstrated a concentration-dependent cytotoxic effect of both AgNPs and ZnONPs. These findings underscore the potential of these green-synthesized nanoparticles for various applications, particularly in fields where their cytotoxic properties can be leveraged. This research serves as a basis for future inquiries regarding the practical utility and risk evaluations of these nanoparticles across various industries.
